# Development of a Triple-Coaxial Flow Device for Fabricating a Hydrogel Microtube and Its Application to Bioremediation

**DOI:** 10.3390/mi9020076

**Published:** 2018-02-12

**Authors:** Kazuma Fujimoto, Kazuhiko Higashi, Hiroaki Onoe, Norihisa Miki

**Affiliations:** 1 School of Integrated Design Engineering, Keio University, 3-14-1 Hiyoshi, Kohoku-ku, Yokohama, Kanagawa 223-8522, Japan; fkazuma2007@gmail.com (K.F.); kazuhiko@z2.keio.jp (K.H.); 2 Department of Mechanical Engineering, Keio University, Yokohama 223-8522, Japan; onoe@mech.keio.ac.jp (H.O.)

**Keywords:** microtubes, triple-coaxial flow, microbes, microfluidics, bioremediation

## Abstract

This paper demonstrates a triple-coaxial flow device to continuously produce a hydrogel microtube using a microfluidic technique. The hydrogel microtube can encapsulate a microbial suspension, while allowing the diffusion of oxygen and nutrients into the microtube and preventing microbes from passing into or out of the microtube. The microtubes also enable the collection of the microbes after task completion without contaminating the environment. In our previous study, we used a double-coaxial flow device to produce the microtubes, but continuous production was a challenge. In the present study, we developed a microfluidic device that fabricates a triple-coaxial flow to enable continuous production of the microtubes. Here, we characterize the production capacity of the microtubes along with their properties and demonstrate bioremediation using microtubes encapsulating a microbial suspension.

## 1. Introduction

Microbes conduct multistep reactions with biological enzymes as catalysts for metabolization [1]. Many of the resulting substances of the metabolism are difficult to produce in vitro; therefore, microbes are currently used in various fields such as food production, medicine, environmental science, and energy [2,3,4,5,6,7]. For practical applications using microbes, the culture system needs to enable mass culture at low cost without biological contamination from competitive microbes.

**Figure 1:**
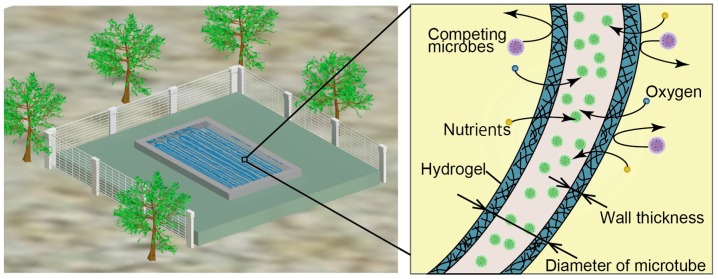
Open culture system with microtubes.

**Figure 2:**
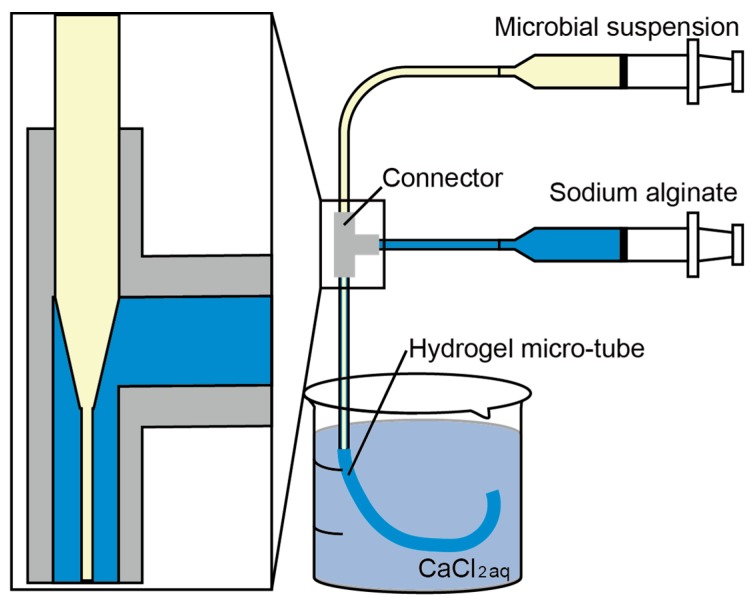
Our previous device with the double-coaxial flow.

In our previous work, we proposed a microbial culture system with hydrogel microtubes, as shown in Figure 1 [8]. The hydrogel tubes are made of calcium alginate, and have pores that are larger than nutrients and oxygen, but smaller than microbes and bacteriophages [9,10]. Therefore, the hydrogel microtubes prevent microbes from passing through the walls while permitting the diffusion of oxygen and nutrients. Target microbes encapsulated inside the tubes are, thus, protected from competing microbes. Moreover, the microbes can be easily collected along with the tubes without contaminating the surrounding environment. We previously developed a microfluidic device to produce the microtubes that encapsulate microbes using double-coaxial flow [11,12,13]. The double-coaxial flow comprises a sodium alginate solution as the outer flow and a microbial suspension as the inner flow. When sodium alginate bonds with calcium ions, it immediately forms a stable three-dimensional gel [14,15]. As shown in Figure 2, the double-coaxial flow flows into the calcium chloride solution and the outer flow, sodium alginate, becomes a hydrogel to form the hydrogel microtube encapsulation. The flow rates and, thus, the production rate, are limited due to Rayleigh–Taylor instability and continuous formation of the tubes is difficult [16,17]. As previously mentioned, mass production is mandatory for practical applications.

In this study, therefore, we developed a triple-coaxial flow device, where the outermost flow is calcium chloride, and hydrogel microtubes emerge from the end of the device. The device enables mass production of the microtubes with negligible Rayleigh–Taylor instability. We first characterize the formation of the hydrogel tubes with respect to the production rate, efficiency, and the tube properties. Then, we demonstrate bioremediation, which is one of the promising microbial applications, using the microtubes with encapsulated microbes. The microtubes were immersed in an aqueous solution of methylene blue to decompose the methylene blue, and when the bioremediation was completed, the tubes and the microbes inside were successfully collected.

## 2. Materials and Methods

### 2.1. Materials

Sodium alginate (80–120 cP) and calcium chloride were purchased from Wako Pure Chemical Industries, Ltd. (Osaka, Japan), and saline was obtained from Otsuka Pharmaceutical Factory (Tokyo, Japan). The concentration of sodium alginate solution was 1.5 *w*/*v*% (7.5 g sodium alginate in 492.5 g deionized (DI) water). The concentration of the calcium chloride solution was 150 mM (16.65 g calcium chloride in 1000 mL DI water). These conditions were found to be appropriate for producing hydrogel microtubes [9,18]. *Coryne glutamicum* (*C. glutamicum*)*, Vibrio alginolyticus* (*V. alginolyticus*)*, Pseudomonas aeruginosa* (*P. aeruginosa*), and *Bacillus subtilis* (*B. subtilis*) were purchased from the National Institute of Technology and Evaluation (Tokyo, Japan) and were used as the target microbes. The culture media of *C. glutamicum, P. aeruginosa*, and *B. subtilis* included 5 g hipolypepton, 1 g yeast extract, 0.5 g MgSO_4_·7H_2_O, and 500 mL DI water. The culture medium of *V. alginolyticus* included 5 g hipolypepton, 1 g yeast extract, 0.25 g MgSO_4_·7H_2_O, 13.35 g Daigo’s artificial seawater SP (NOVA Chemicals, Calgary, Canada), and 500 mL DI water. All the materials for producing the different culture media were purchased from Wako Pure Chemical Industries. Methylene blue aqueous solution was used to demonstrate bioremediation [19,20], and was prepared by mixing 0.029 g methylene blue trihydrate powder (Hayashi Pure Chemical Ind., Ltd., Osaka Japan) and 500 mL DI water. The solution for the experiments of the removal of methylene blue dye included 15 mL methylene blue solution, 50 mL DI water, and 20 mL culture medium

### 2.2. Triple-Coaxial Flow Device

**Figure 3:**
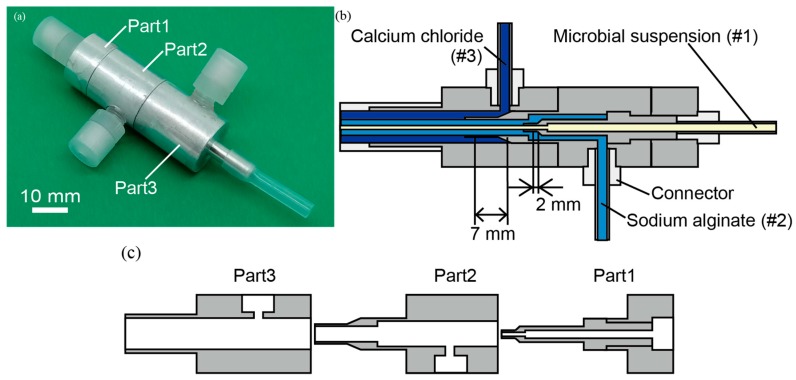
Triple-coaxial flow device. (**a**) Image of the device. Cross-sectional view of (**b**) the device and (**c**) the three parts to produce the triple-coaxial flow.

A photo and the cross-sectional view of the triple-coaxial flow device are shown in Figure 3. The device consists of three aluminum parts made using a lathe and numerically-controlled milling machine (Figure 3a). As shown in Figure 3b,c, the triple-coaxial flow consists of three fluids: (#1) the microbial suspension, (#2) the sodium alginate solution, and (#3) the calcium chloride solution. A hydrogel microtube encapsulating the microbial suspension is produced by the formation of the triple-coaxial flow from the three fluids, and emerges from the outlet of the device. To observe the effect of the outlet diameter, we designed separate Part 2 sections of the device with three different outlet diameters.

**Figure 4:**
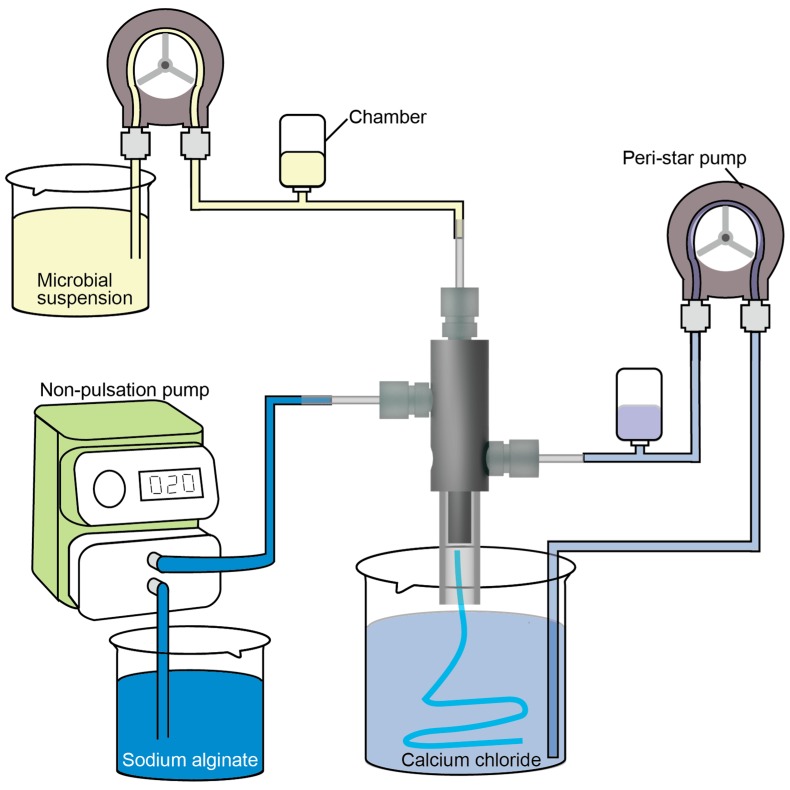
Experimental setup for production of microtubes.

The experimental setup is shown in Figure 4. The triple-coaxial flow device was fixed to a stand. The microbial suspension was introduced into the device using a liquid delivery pump (Peri-star pump Pro, Tacmina Co., Osaka, Japan). The sodium alginate solution was introduced into the device using a pulseless pump (Smoothflow pump Q, Tacmina Co., Osaka, Japan). The calcium chloride solution was introduced into the device using a liquid delivery pump (Masterflex L/S, Yamato Scientific Co, Ltd., Tokyo, Japan). Each pump and the device were connected using silicone tubing. Pulsatile flow prevents a stable formation of the triple-coaxial flow, so an air chamber was placed between the pumps and the device to reduce the pulsations generated by the liquid delivery pumps. When we stop producing the microtube, #1, #2, and #3 flows are stopped in turn.

### 2.3. Fabrication of a Hollow Hydrogel Microtube

Saline dyed with red stain was used as fluid #1 instead of a microbial suspension to characterize the formation of the microtubes. We investigated the wall thickness of the microtube with respect to the volumetric flow rates of fluids #1 and #2. The volumetric flow rate of fluid #1 was set from 12.5 to 27.5 mL/min, the volumetric flow rate of fluid #2 was set from 25 to 35 mL/min, and the volumetric flow rate of fluid #3 was kept constant at 75 mL/min. Fluids #3, #2, and #1 were sequentially introduced into the device. The microtubes were subsequently collected in the calcium chloride solution. The outer and inner diameters of the fabricated microtubes were measured at three locations using an optical microscope (VHX-600, KEYENCE, Osaka, Japan). 

### 2.4. Bioremediation: Removal of Methylene Blue Dye

First, we conducted preliminary experiments to determine the suitable microbes for bioremediation. We used methylene blue to visualize the ability of the microbes to decompose organic matter. The tested microbes included *C. glutamicum*, *V. alginolyticus*, *P. aeruginosa*, and *B. subtilis* [21,22,23]. To investigate the capability of these microbes to decompose methylene blue, 15 mL of each microbial suspension was added to a solution consisting of 15 mL methylene blue solution and 70 mL DI water. To evaluate the degree of methylene blue decomposition, we measured changes in the absorbance of methylene blue with a spectrophotometer (UV3600, Shimadzu, Kyoto, Japan) after one day, four days, seven days, and 14 days. The absorbance was measured three times for each solution.

Next, we produced a hydrogel microtubes encapsulating 15 mL of the microbial suspension of *B. subtilis* following the results of the preliminary tests. The produced microtube was immersed into the methylene blue solution. After one day, four days, and seven days, the absorbance and the degree of the turbidity of the methylene blue aqueous solution was measured three times using the spectrophotometer. We also attempted to collect *B. subtilis* along with the microtubes to confirm that collection of encapsulated microbes is possible without contaminating the solution with the microbes.

## 3. Results and Discussion

### 3.1. Fabrication of a Hollow Hydrogel Microtube

**Figure 5:**
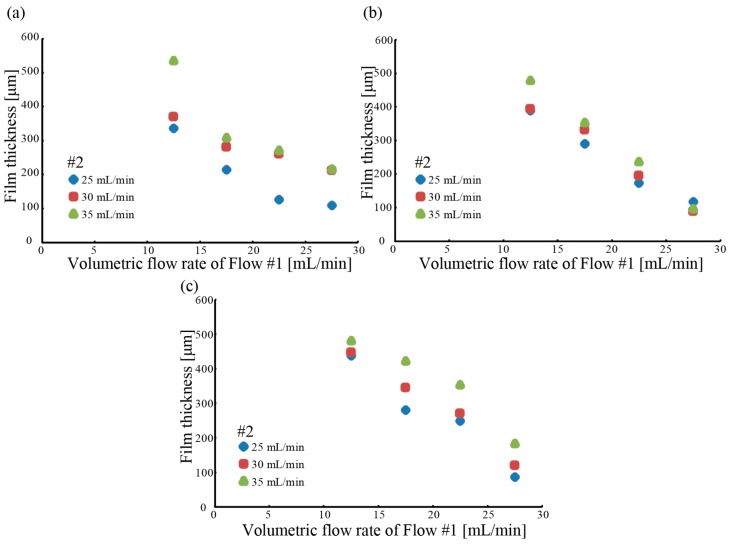
Relationship between film thickness of microtube and volumetric flow rates of fluids #1 and #2. (**a**) Outlet diameter of Part 2 is 2.5 mm. (**b**) Outlet diameter of Part 2 is 2.7 mm. (**c**) Outlet diameter of Part 2 is 2.9 mm.

**Figure 6:**
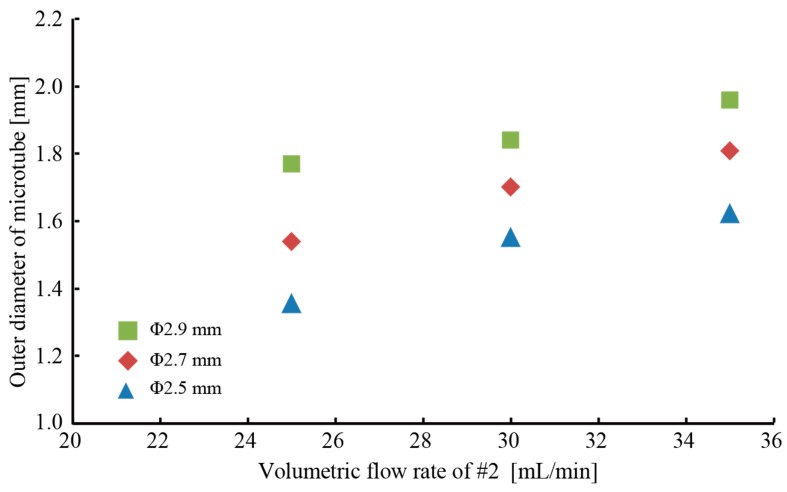
Relationship between outer diameter of microtube and volumetric flow rate of fluid #2.

**Figure 7:**
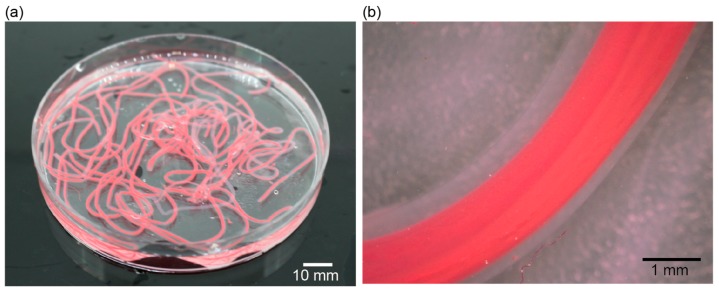
(**a**) Photo of the produced hydrogel microtube with the triple-coaxial device. (**b**) Photomacrogragh of the hydrogel microtube.

Figure 5 shows the film thickness of the produced microtube with respect to the volumetric flow rate. As the volumetric flow rate of fluid #1 increased, the inner diameter of the microtube increased, but the film thickness decreased. It was also found that the film thickness increased as the volumetric flow rate of fluid #2 increased. Thereupon, it was found that the film thickness was controlled from 100 to 500 μm. We investigated the effect of the outlet diameter of Part 2. Figure 6 shows the outer diameter of the microtube produced by the triple-coaxial flow device with respect to the volumetric flow rate of fluid #2. The microtube diameter increased as both the outer diameter of Part 2 and the volumetric flow rate of fluid #2 increased. Furthermore, it was found that the diameter of the hydrogel microtube was controlled from 1.4 to 2.0 mm. In our previous study we found that, to ensure diffusion of nutrients and oxygen through the tube wall, the wall thickness needed to be less than 250 μm. Moreover, for mass culture of target microbes, the diameter of the microtube should be as large as possible. Therefore, we chose volumetric flow rates of 27.5, 35, and 75 mL/min for fluids #1, #2, and #3, respectively. This is because of the produced microtube at the volumetric flow rates. A photo of the produced hydrogel microtube encapsulating dyed saline is shown in Figure 7.

**Figure 8:**
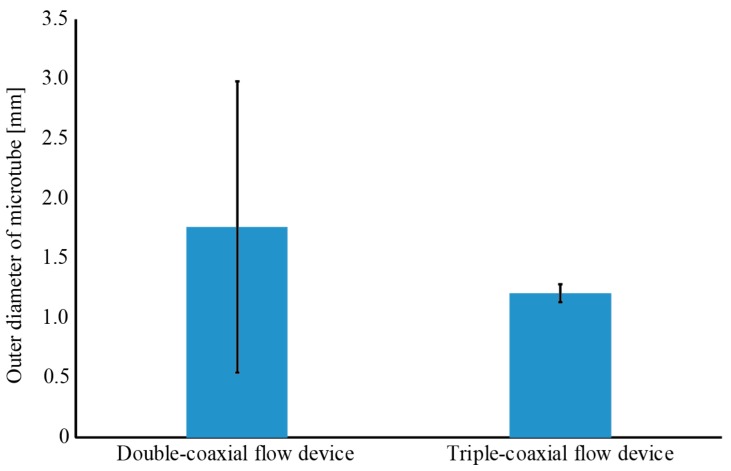
Comparison of outer diameters of microtubes devices produced using the double- and triple-coaxial flow.

Microtubes were produced at a rate of 281 mm/s. This is approximately 25 times faster than the 11 mm/s achieved in our previous study using a double-coaxial flow device. Next, we measured the outlets at 10 locations on microtubes produced using double- and triple-coaxial flow devices. Figure 8 shows the average diameter and the standard error of the microtubes produced using the two devices. As we described in the Introduction, the Rayleigh–Taylor instability generated during fabrication of the microtubes using the double-coaxial flow device results in the formation of a hydrogel mass. Therefore, the standard error of the outlet diameter of the microtubes was large because of the formation of this hydrogel mass. The triple-coaxial flow can produce the hydrogel tubes with sufficient mechanical strength before they reach the calcium chloride solution, or collecting solution. These differences resulted in higher production rates and smaller deviations in the tube diameter.

### 3.2. Removal of Methylene Blue Dye

**Figure 9:**
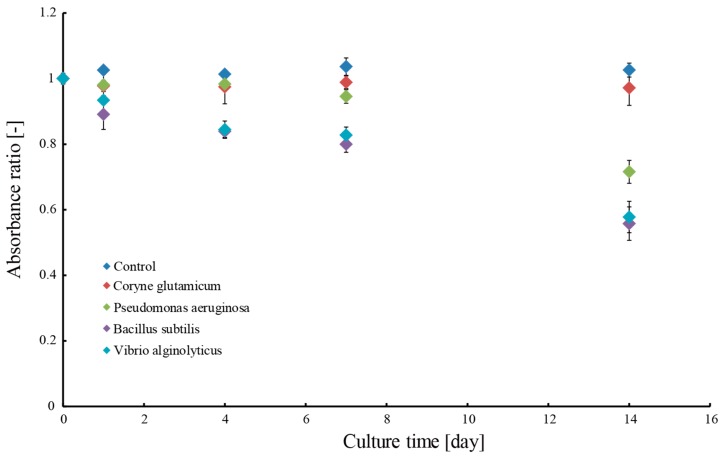
Relationship between absorbance ratio of methylene blue and culture time of each microbe.

Figure 9 shows the degree of decomposition of methylene blue for each of the target microbes. *V. alginolyticus*, *P. aeruginosa*, and *B. subtilis* decomposed the methylene blue, while *C. glutamicum* did not. Among the tested microbes, *B. subtilis* exhibited the highest performance in decomposition of methylene blue. Based on the results of this experiment, *B. subtilis* could have reduced the concentration of methylene blue in the aqueous solution by 46% in two weeks. Therefore, we decided to use *B. subtilis* as the target microbe in further experiments testing the effectiveness of encapsulated microbes. Notably, the standard error of the absorbance ratio for each microbe increased as the culture time progressed. This is because the turbidity caused by the increase in the number of microbes interferes with the absorbance measurements of methylene blue.

**Figure 10:**
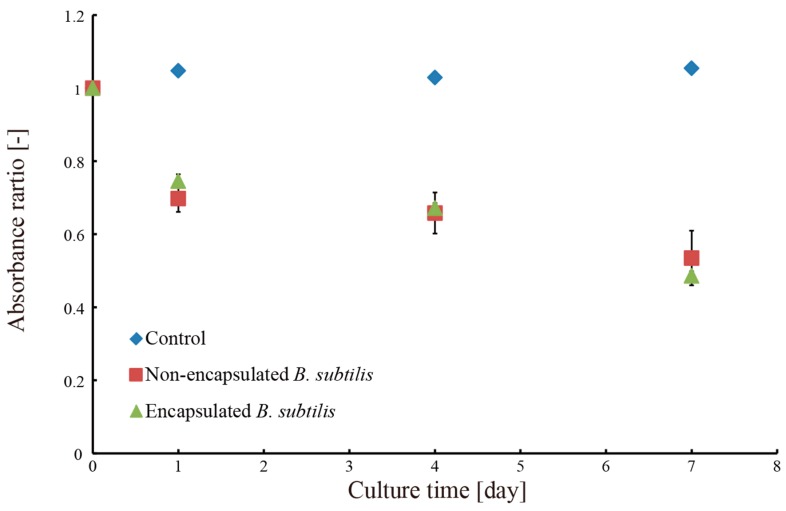
Relationship between the absorbance ratio of methylene blue and culture time with encapsulated and non-encapsulated *B. subtilis.*

Next, we produced microtubes encapsulating a microbial suspension of *B. subtilis*. The produced microtubes were immersed into the methylene blue aqueous solution. Figure 10 shows the degree of decomposition of methylene blue using the hydrogel microtubes. The hydrogel microtubes encapsulating the microbial suspension decomposed methylene blue at a comparable rate to the microbes that were not encapsulated. It is considered that *B. subtilis* encapsulated in the hydrogel microtube can obtain enough oxygen and nutrients.

**Figure 11:**
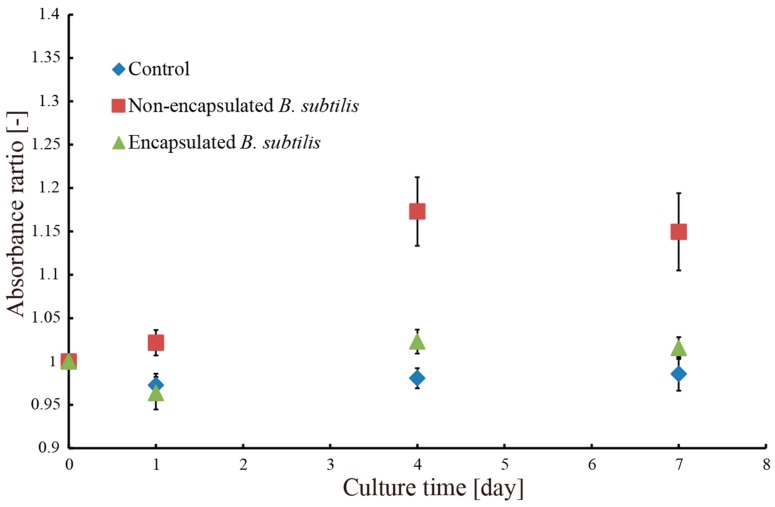
Relationship between the concentration of microbes in the methylene blue solution and culture time.

Figure 11 shows the opacity of the methylene blue aqueous solution during the bioremediation experiments by measuring the changes in the absorbance ratio of the methylene blue aqueous solution. When *B. subtilis* was introduced into the solution, the opacity of the solution increased due to the presence of the microbes. However, when the microtubes containing the microbes were introduced into the solution, the value of the absorbance ratio of the concentration of microbes had fallen in the range 0.95–1.05. The absorbance ratio of the control solution, which did not contain microbes, had fallen in the same range. Therefore, the opacity did not increase. This indicates that the microbes did not leak out of the microtubes. Again, the higher standard error of the absorbance ratio was caused by the turbidity of the solution containing *B. subtilis* not encapsulated in the microtubes. Furthermore, the microtubes were successfully collected and removed from the solution along with the microtubes, which prevented the contamination of the solution by the microbes. This can only be achieved by encapsulating the microbes in the microtubes. As shown in Figure 10, the encapsulated microbes could decompose the methylene blue. *B. subtilis* encapsulated in the hydrogel microtubes reduced the concentration of the methylene blue solution by 50% in seven days.

## 4. Conclusions

We developed and characterized a triple-coaxial flow device to produce hydrogel microtubes continuously at a high rate. The diameter and the film thickness of the hydrogel microtubes were successfully controlled with the volumetric flow rates of the fluids and the diameter of the channel. Therefore, by using the triple-coaxial flow device, the diameter of the hydrogel microtube was controlled from 1.4 mm to 2.0 mm, and the film thickness was controlled from 100 μm to 500 μm. The triple-coaxial flow device was able to produce microtubes 25 times faster than our previously-proposed double-coaxial flow device. By reducing the effect of Rayleigh–Taylor instability, the produced hydrogel microtubes had smoother features and less deviation in their diameter. The effectiveness of the hydrogel microtubes encapsulating microbes was demonstrated in bioremediation experiments. *B. subtilis* encapsulated inside the microtubes successfully decomposed methylene blue and did not leak out of the tubes, thereby preventing contamination of the solution by the microbes. Moreover, the microbes were easily collected from the solution along with the microtubes.
